# Type B choledochocele vs duodenal duplication cyst: a diagnostic dilemma and its management: a case report

**DOI:** 10.1186/s13256-019-2010-2

**Published:** 2019-05-24

**Authors:** M. KarthiKeyan, L. SoundaraRajan, M. Karthi, M. UmaMaheswaran, S. Rajendran

**Affiliations:** Institute of Surgical Gastroenterology, Rajiv Gandhi Government General Hospital, Madras Medical College, Chennai, India

**Keywords:** Duplication cyst, Choledochocele, Case report, Marsupialization, Gut signature, Ampulla

## Abstract

**Introduction:**

Duplication cyst of the alimentary tract is a rare congenital anomaly. Duodenal duplication cyst accounts for less than 5% overall. These entities rarely present in adults. They are often mistaken as choledochoceles. Management is most often complete excision, but it is individualized to the particular case.

**Case presentation:**

A 22-year-old woman was admitted to our hospital with a history of intermittent colicky right hypochondrial pain not relieved by any medications for the past 3 months. Initially, she was given proton pump inhibitors, but her pain was not relieved. Further evaluation was done, and preoperative imaging showed a cyst in the second part of the duodenum. Magnetic resonance imaging revealed it as a choledochocele, but duodenal duplication cyst was kept in the differential diagnosis. Further ultrasound identified it to be a duplication cyst. After failed endotreatment, the patient was successfully managed with partial excision and marsupialization.

**Conclusion:**

Duodenal duplication cyst is uncommon and rarely diagnosed in adults. Duplications in the duodenum should always be a part of the differential diagnosis, especially in cystic lesions. Ultrasonogram of the cyst might lead to the proper diagnosis. Surgery is the treatment of choice if endotherapy is not successful.

## Background

Alimentary tract duplications are a rare congenital anomaly that can occur anywhere from the oropharynx to the anus. Duplication cyst is a spherical or tube-shaped structure lined by luminal epithelial cells. Duodenal duplications account for about 6% of alimentary tract duplications. Prevalence of duplications is about 1 in 100,000 live births. Duplication cyst in the duodenum often was mistaken as choledochocele, which was the initial diagnosis in our patient. Further workup with a multidisciplinary team led to the correct diagnosis and management.

## Case presentation

**Fig. 1:**
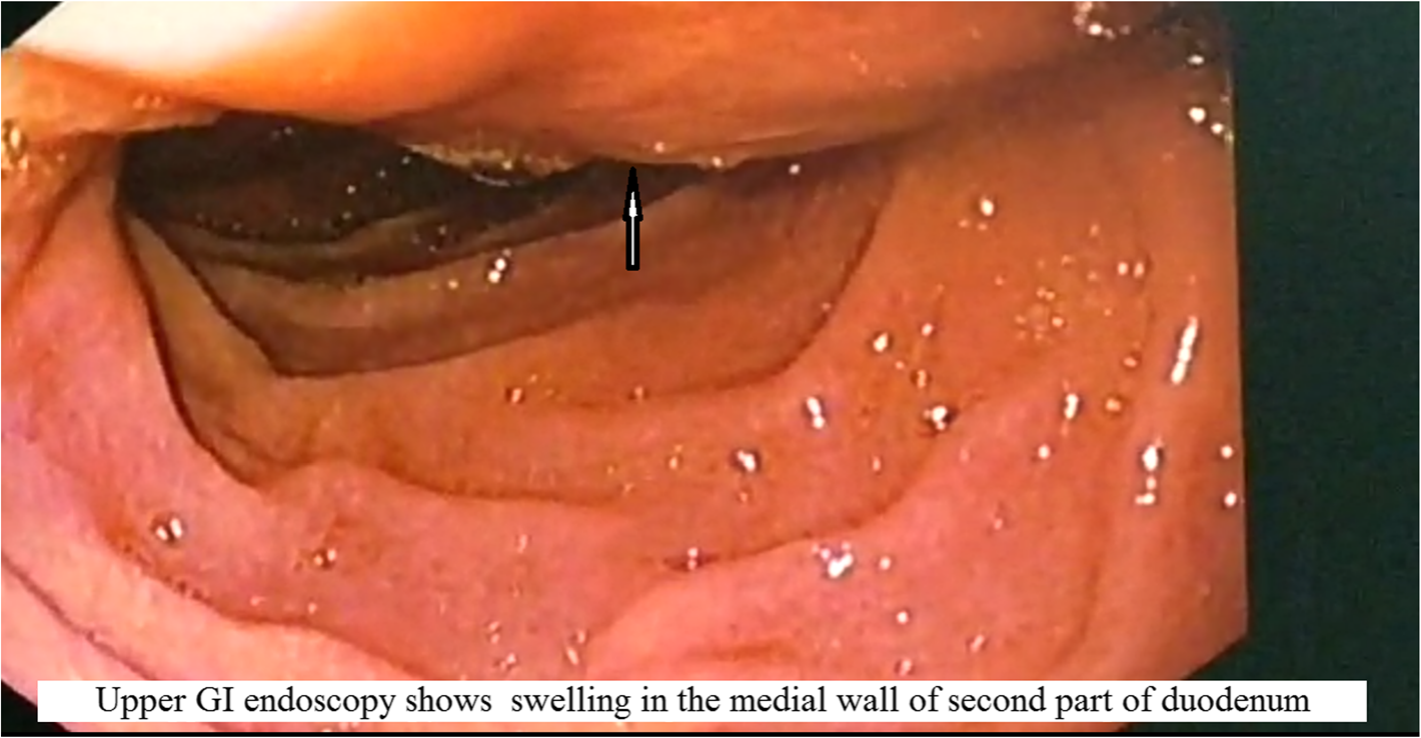
Upper GI endoscopy showing swelling (arrow) in the medial wall of the second part of duodenum

A 22-year-old woman was admitted to our hospital with a history of intermittent colicky right hypochondrial pain not relieved by any medications for the past 3 months. Initially, she was given proton pump inhibitors, but her pain was not relieved. She had no other symptoms. Her past medical and family histories were not significant. The result of her clinical examination was normal. Upper gastrointestinal (GI) endoscopy showed globular swelling at the medial wall of D2 (Fig. 1). The ampulla was situated at the summit of swelling.

**Fig. 2:**
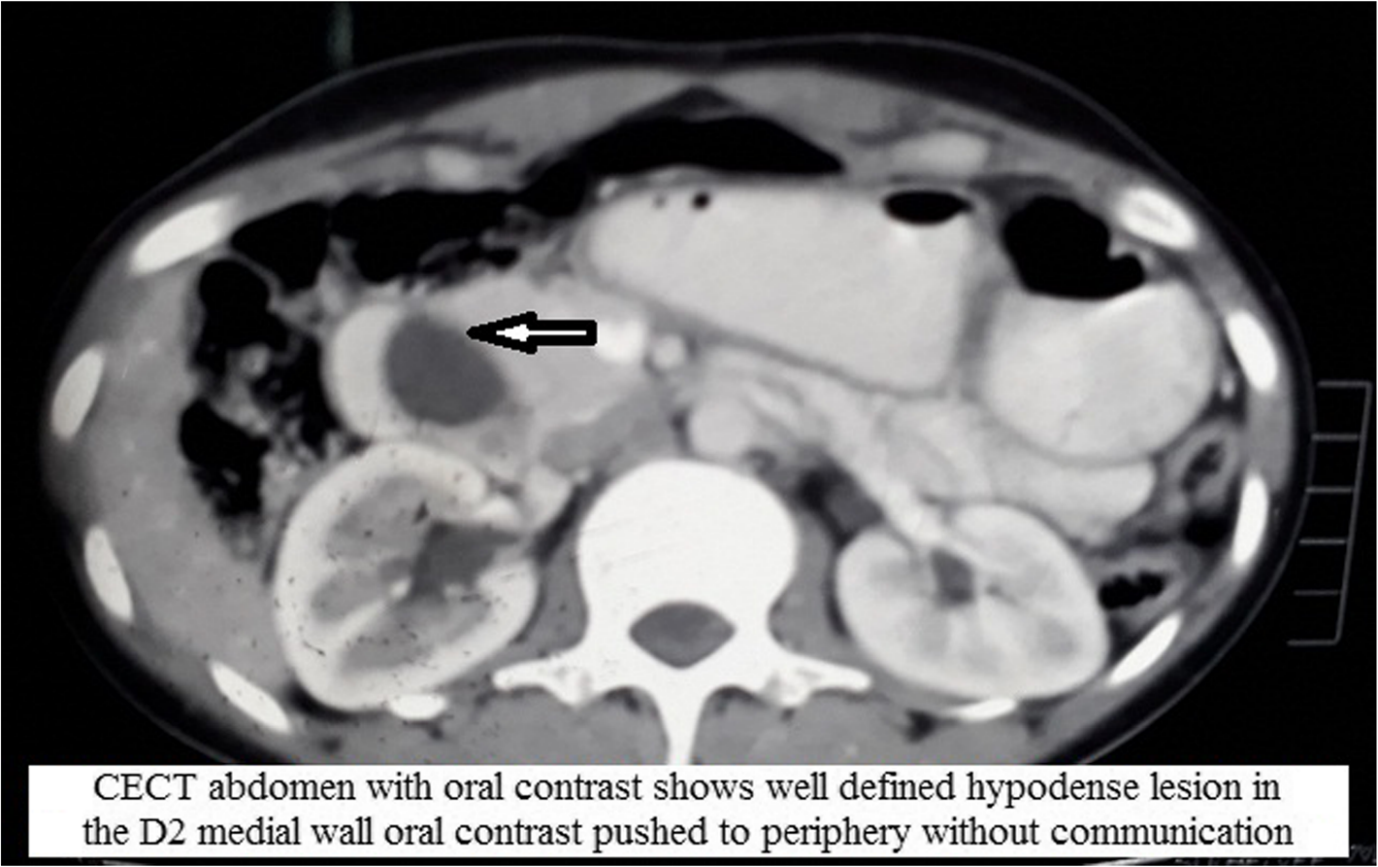
CECT abdomen with oral contrast shows well defined hypodense lesion (arrow) in the D2 medial wall oral contrast pushed to periphery without communication

**Fig. 3:**
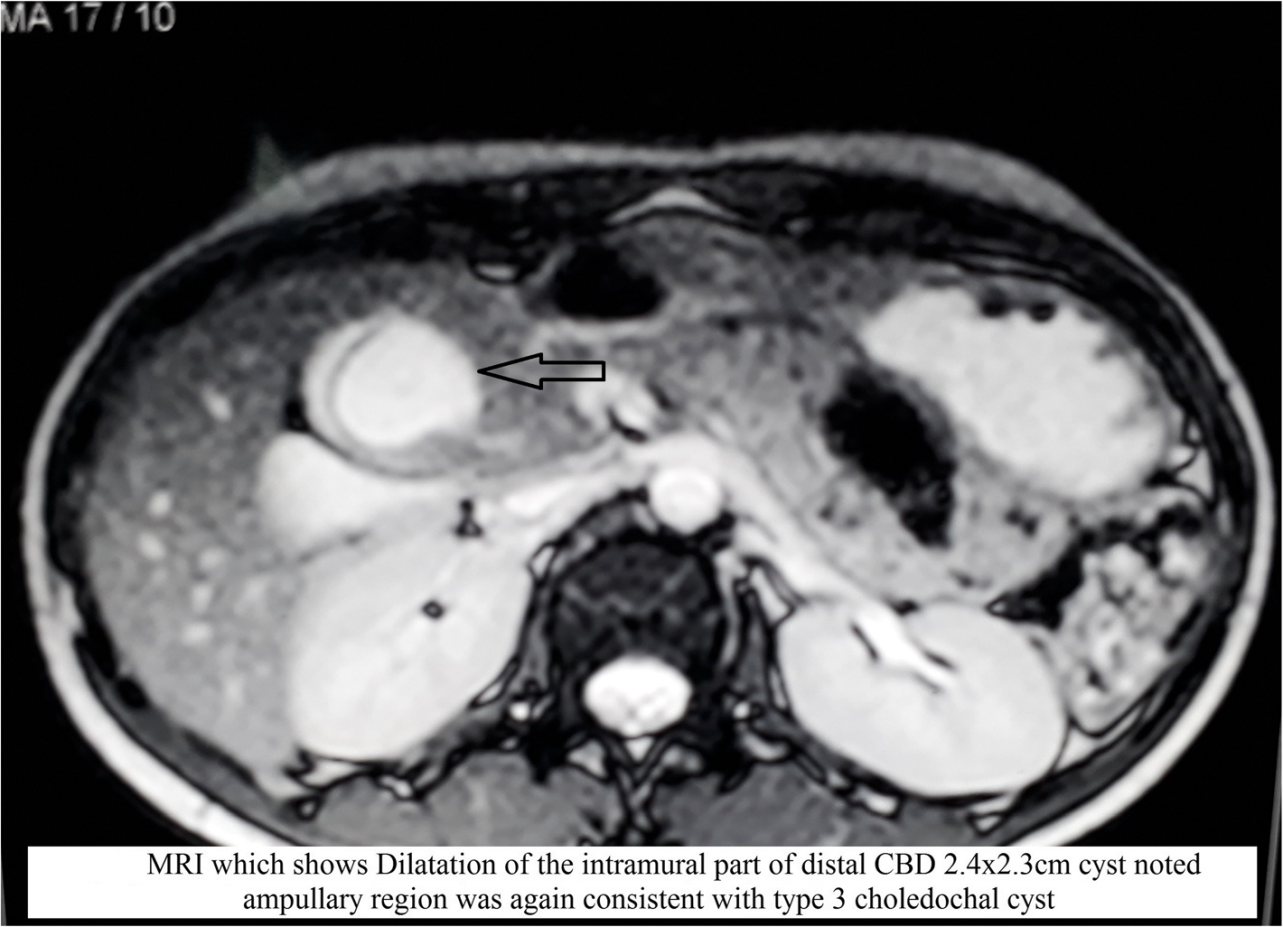
MRI abdomen showing dilatation of the intramural part of distal CBD 2.4 x 2.3cm cyst (*arrow*) noted ampullary region suggestive of Type 3 choledochal cyst

**Fig. 4:**
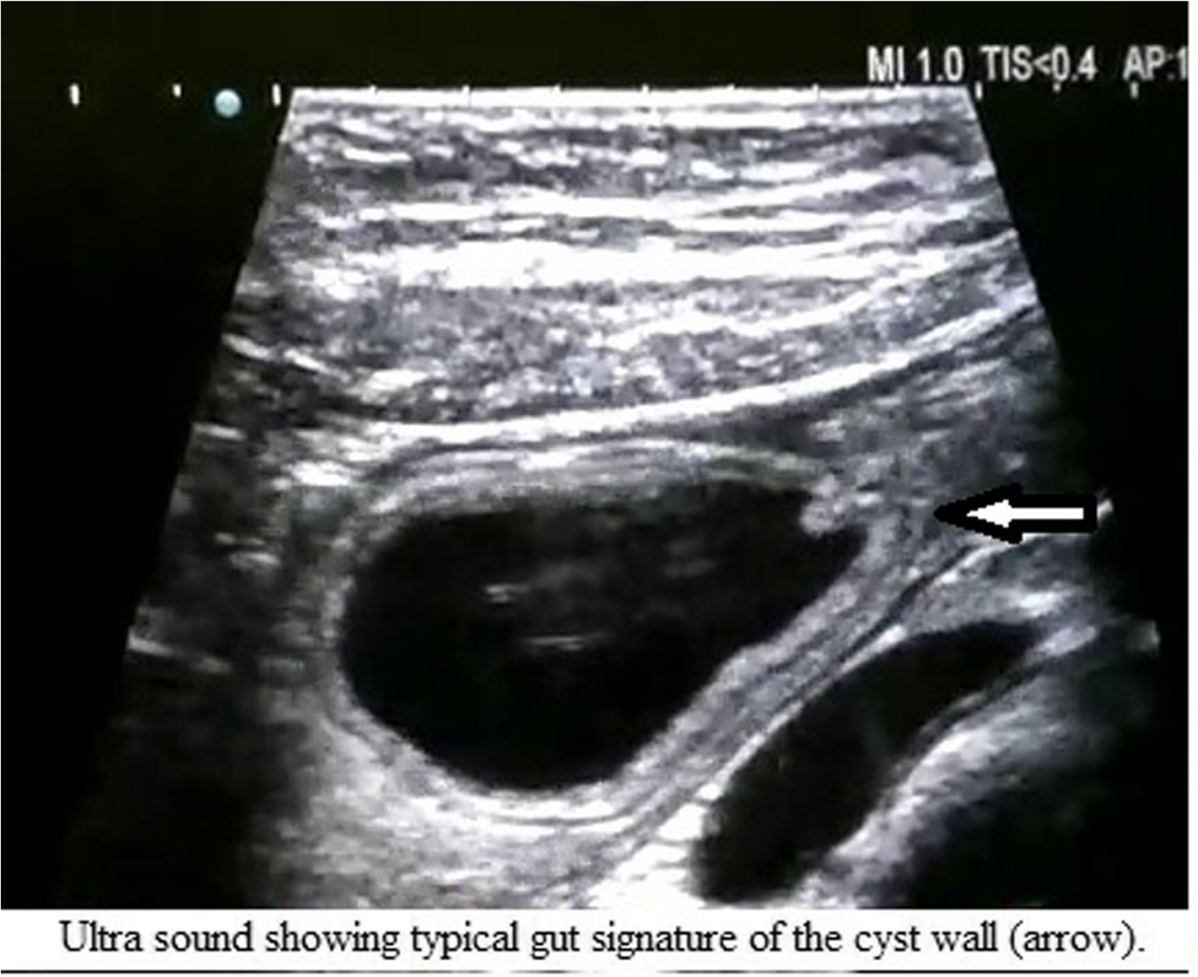
Ultra sound showing typical gut signature of the cyst wall (*arrow*)

Computed tomography (CT) with oral contrast agent showed dilation of the intramural part of the common bile duct (CBD). A cyst of size 2.4 × 2.3 cm was noted in the second part of the duodenum (Fig. 2). On the basis of the above findings, it was reported as type 3 choledochal cyst. The patient was further investigated with magnetic resonance imaging, which showed dilation of the intramural part of the distal CBD. A 2.4 × 2.3 cm cyst was noted in the ampullary region, again consistent with a type 3 choledochal cyst (Fig. 3). Then the patient was planned for endotherapy, but owing to difficulty, it could not be negotiated into the ampulla. Finally, the patient was referred to the surgical gastroenterology department for operative management. After multidisciplinary team discussion, an abdominal ultrasonogram (USG) was done, which showed a clear thick wall cyst measuring 4.6 × 2.6 cm between the second part of the duodenum and the head of the pancreas with gut signature (Fig. 4). Opening of the CBD into the duodenum was seen just distal to the cystic lesion. There was active peristalsis noted all around the cyst, suggestive of duodenal duplication cyst.

**Fig. 5:**
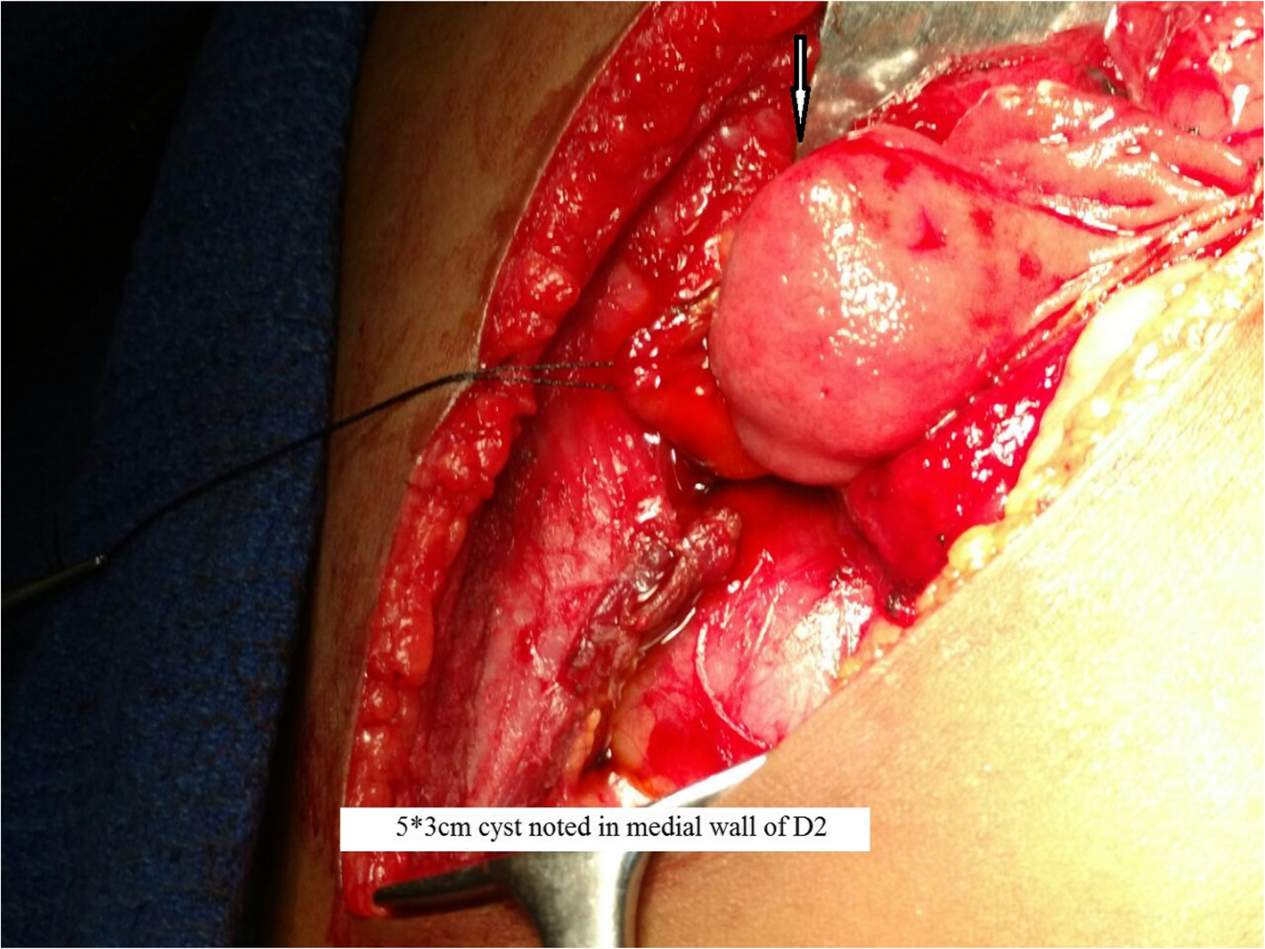
Intra operative image showing 5 x 3cm cyst (arrow) noted in medial wall of D2

**Fig. 6:**
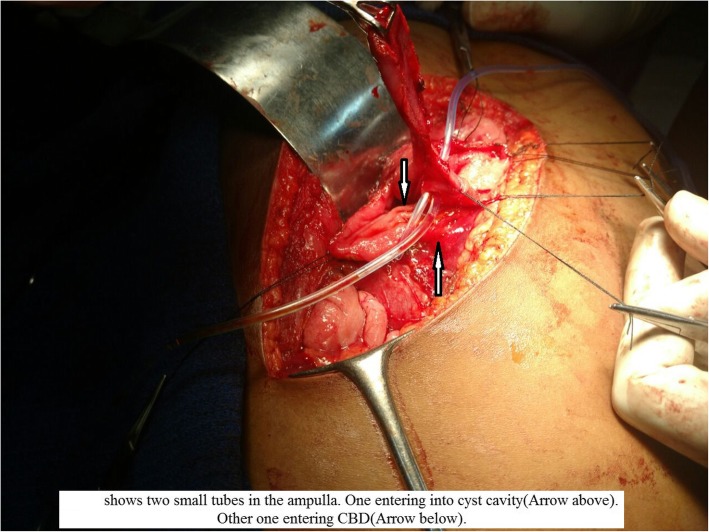
Intraoperative image showing two small tubes in the ampulla. One entering into cyst cavity (*Arrow* above). Other one entering CBD (*Arrow* below)

**Fig. 7:**
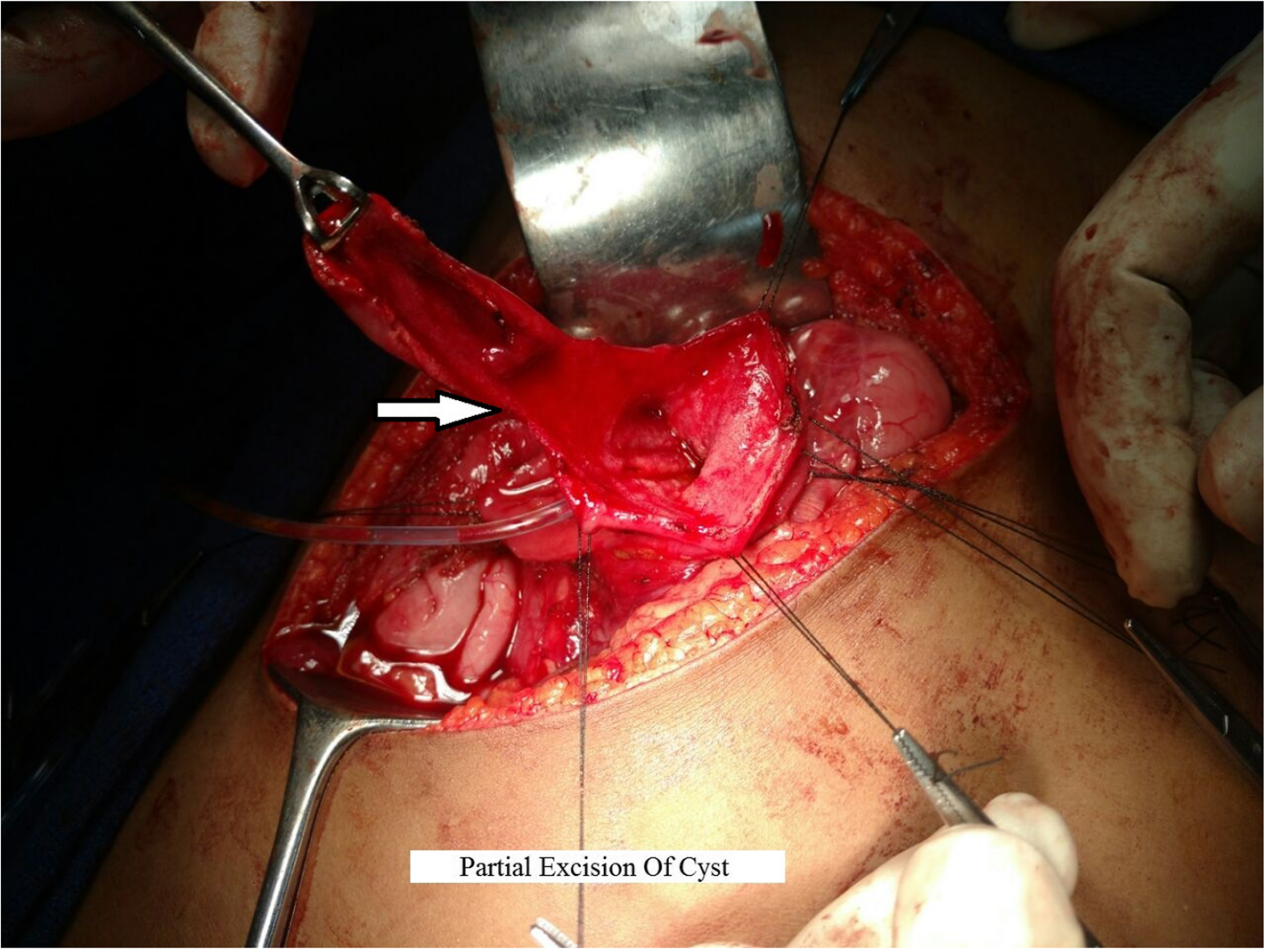
Intraoperative image showing partial excision (arrow) of the cyst

**Fig. 8:**
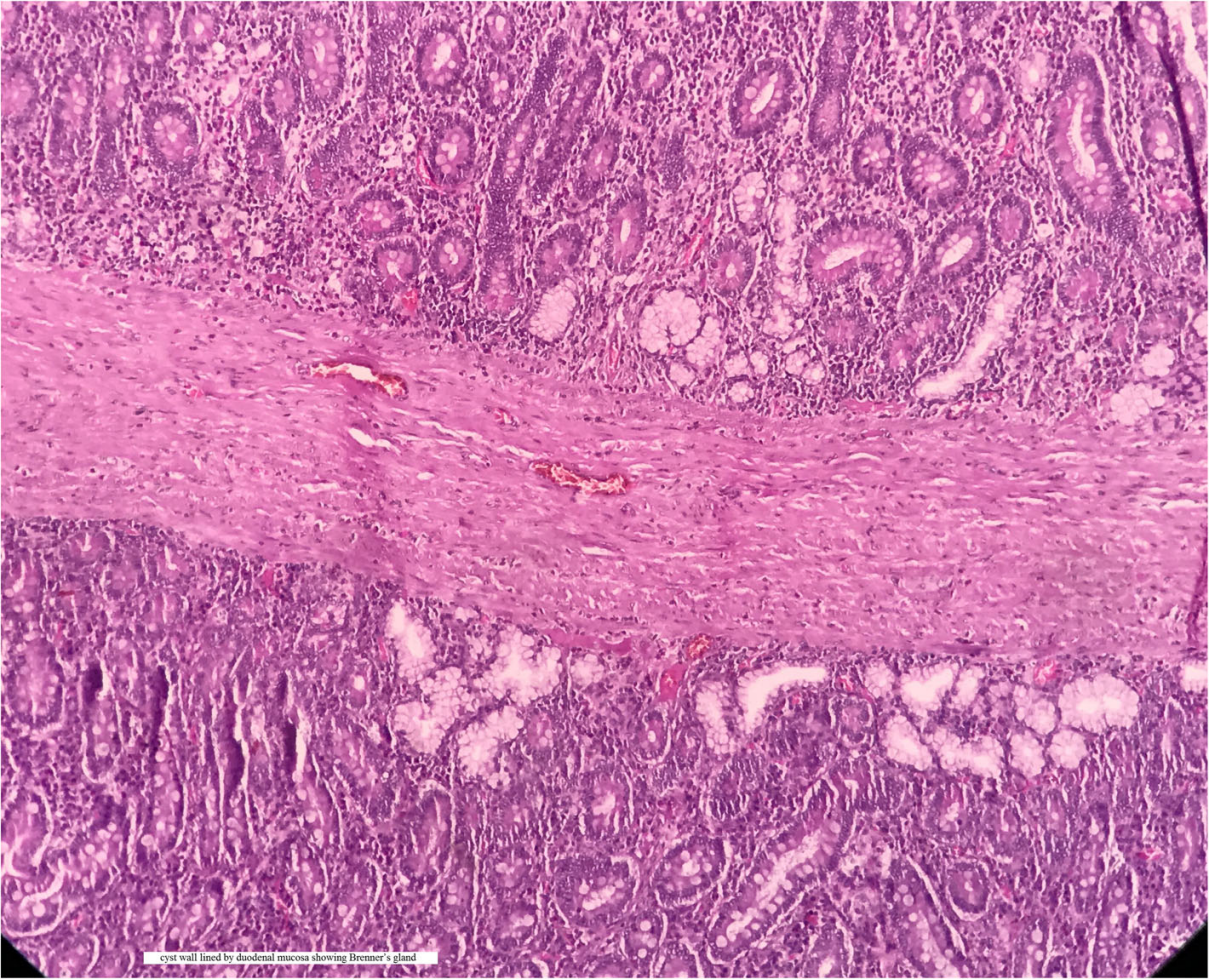
Histopathological Section showing cyst wall lined by duodenal mucosal epithelium with focal areas of ulceration and composed of tall columnar cells with goblet cells on either side of a common (shared) muscular layer. Submucosa shows lymphoid aggregates with brunner glands. The common muscular layer shows congested vessels

After the diagnosis was confirmed, the patient was taken for elective laparotomy. Intraoperative findings were a 5 × 3-cm cyst over the medial wall of the duodenum extending proximal and distal to the ampulla and displacing it posteriorly (Fig. 5). The cyst was communicating with the ampulla by a small opening in its medial wall (Fig. 6). So, cyst secretions were drained via the ampulla, which avoided retention of cystic fluid. Part of the cyst wall was shared with duodenal musculature. Upon needle aspiration, the cyst wall contained bile due to communication with the ampulla. The duodenum was Kocherized, and a longitudinal duodenotomy was made for about 5 cm. Partial excision of the cyst was done (Fig. 7) because it was closely associated with the ampulla. Marsupialization of the remaining cyst wall was done. The duodenotomy was closed horizontally. Feeding jejunostomy (FJ) was done using a modified Witzel method. A flank drain was kept in place. The postoperative period was uneventful. The patient was started on oral medications on the third day after surgery. The FJ tube was removed after 6 weeks. Histopathology showed the cyst wall was lined by duodenal mucosal epithelium with focal areas of ulceration and composed of tall columnar cells with goblet cells on either side of a common (shared) muscular layer. The submucosa showed lymphoid aggregates with Brunner glands. The common muscular layer showed congested vessels. Histopathological features were suggestive of duodenal duplication cyst (Fig. 8). At her 9-month follow-up visit, the patient had no complaints.

## Discussion

**Fig. 9:**
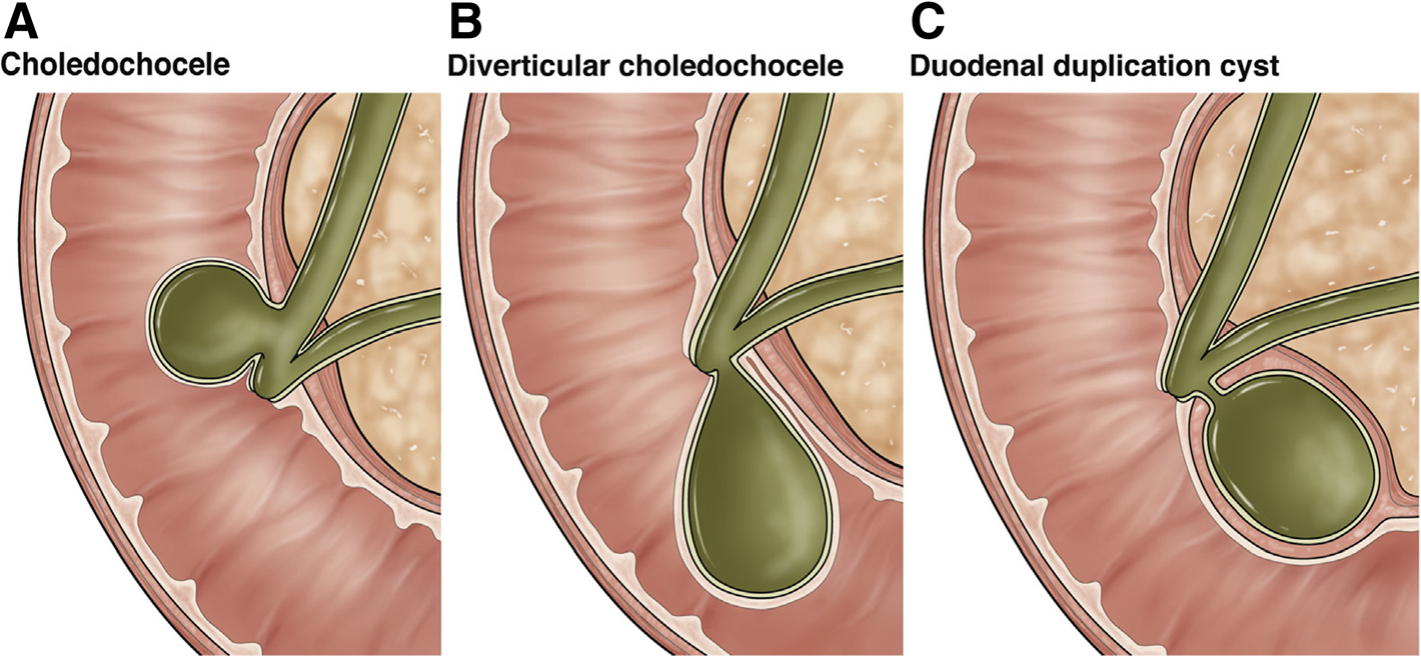
**a** shows type A choledochocele, **b** shows type B choledochocele and **c** shows Duodenal duplication cyst. See that B and C communicating to the ampulla. C covered with muscle coat that differentiate this from the Type B choledochocele (copy rights obtained from Elsevier-reference 11)

Duodenal duplication cyst is a rare congenital anomaly [1]. Presentation in adults is infrequent. These cysts are often mistaken for type 3 choledochal cysts that are choledochoceles. Choledochocele is a more frequent cystic lesion in the duodenal region than a duplication cyst. Choledochocele presents at an older age, with the average age at presentation being 51 years. Owing to proximity to the ampulla and the second part of the duodenum, choledochocele and duplication cyst share common features, such as location, communication with the ampulla (*see* Fig. 9), and epithelial lining by duodenal mucosa, but choledochocele is also lined by biliary epithelium [2].

The duplication cyst is confirmed by a smooth-muscle coat, an alimentary epithelial lining, and an intimate attachment to the GI tract. It presents as a cystic or tubular communicating or noncommunicating structure to the adjacent alimentary tract. In 1937, William E. Ladd coined the term “duplications of alimentary tract.” Gross *et al.* pointed out the features as the following: (1) the presence of smooth muscle coat, (2) an epithelial lining representing some type of intestinal tract mucosa, and (3) intimate anatomic association with some portion of the alimentary tract [3].

Duplication cysts are usually located at the first and second parts of the duodenum, and the mesenteric side of the anterior wall is becoming more common. Submucosal and intermuscular sites are also described [4, 5]. They can extend into anterior and posterior sides. Most duodenal duplications are diagnosed in childhood, and cysts detected later in life are an extremely rare finding. They can present as palpable masses in the abdomen, especially in children [6], with signs of intestinal obstruction and abdominal pain. Ectopic gastric mucosa can appear and cause ulceration, bleeding, and even perforation. Pancreatitis [7] and rarely malignancy [8] are also reported. The preoperative diagnosis of intestinal duplications is rarely accurate. The classical “gut signature” of its wall observed by abdominal or endoscopic USG and peristalsis of the cyst wall noted upon real-time USG are strongly suggestive of a duplication cyst [3, 9]. Barium studies show mass effect and displacement of normal alignment and details about communication. CT of the abdomen is valuable in identifying the type, location, and size of the duplication cyst [5].

Ideal treatment for duplication cysts is a complete surgical resection. Patients may require pancreaticoduodenectomy when the cyst is close to the ampulla of Vater. Therapeutic endoscopy [10], such as endoscopic mucosal resection and endoscopic submucosal dissection, provides an alternative viable option. Pancreaticoduodenectomy for a benign lesion, considering its morbidity, makes it a last option. Because endotherapy could not be performed successfully, our patient underwent partial cyst excision and marsupialization. Histopathology of duplications will have a classical smooth muscle covering with intestinal epithelial lining and anatomic location adjacent to the GI system. In 9 months of follow-up, our patient was doing well. This case is presented for its rarity, the difficulties we had in preoperative diagnosis, and management with partial excision and marsupialization.

## Conclusion

Duodenal duplication cyst is extremely rare, and it is rarely diagnosed in adults. Duplications in the duodenum should always be a part of the differential diagnosis in cystic lesions. Choledochocele is frequently encountered and should be differentiated from duplications. Endotherapy is the preferred treatment and first line in management. Failed endoscopic treatment necessitates surgery.

## Abbreviations

CBD: Common bile duct
CT: Computed tomography
FJ: Feeding jejunostomy
GI: Gastrointestinal
USG: Ultrasonogram
